# Dietary D-Ribose Supplementation in Sheep: Implications on Rumen, Fecal Microbiota, and Metabolic Function

**DOI:** 10.3390/microorganisms13112505

**Published:** 2025-10-31

**Authors:** Qinghua Qiu, Lin Li, Ke Pan, Kehui Ouyang, Mingren Qu, Huan Liang

**Affiliations:** Jiangxi Province Key Laboratory of Animal Nutrition and Feed, College of Animal Science and Technology, Jiangxi Agricultural University, Nanchang 330045, China

**Keywords:** fecal microbiota, microbial biomarker, nutritional intervention, rumen microbiota, ruminant production, sheep

## Abstract

The objective of this study was to investigate the effects of dietary D-ribose supplementation on the microbial diversity, community composition, and metabolic function of the rumen and fecal microbiota in Hu sheep. Eighteen sheep with similar body weights (20.47 ± 0.58 kg) were selected and randomly divided into two groups, with nine sheep in each group. One group was fed a basal diet (Control), while the other group was supplemented with 300 mg kg^−1^ of D-ribose in addition to the basal diet (D-Ribose). The results showed that D-ribose supplementation had no significant effect on the richness, diversity, or evenness of the rumen and fecal microbiota (*p* > 0.05). D-ribose supplementation lowered the relative abundance of Cyanobacteria in the rumen while increasing that of *Herbivorax* and *Faecalibacterium* (*p* < 0.05). In feces, it decreased the relative abundances of Verrucomicrobia, *Candidatus* Saccharibacteria, *Bifidobacterium*, and *Caproiciproducens*, while increasing that of *Lawsonibacter* and *Massilioclostridium* (*p* < 0.05). Non-metric multidimensional scaling (NMDS) analysis of the rumen microbiota revealed a significant overlap between the Control and D-Ribose groups, and analysis of similarities (ANOSIM) showed no significant differences between the two groups (R = 0.079, *p* = 0.115). In contrast, NMDS analysis of the fecal microbiota showed partial overlap between the two groups, and ANOSIM indicated a significant difference between the Control and D-Ribose groups (R = 0.203, *p* = 0.017). Dietary D-ribose supplementation had no significant effect on any metabolic function with relative abundance greater than 1% in both the rumen and fecal microbiota (*p* > 0.05). The results indicated that dietary D-ribose supplementation did not affect the microbial diversity and metabolic function of the rumen and fecal microbiota but altered the relative abundances of certain bacterial genera. This study provides a perspective on rumen and fecal microbiota to more comprehensively evaluate the effects of dietary D-ribose supplementation on ruminants and offers data support for the application of D-ribose in ruminant production.

## 1. Introduction

In the intricate landscape of ruminant nutrition, optimizing growth performance is a complex challenge that hinges on the delicate balance between feed intake, nutrient absorption, and metabolic efficiency [1]. Feed additives have emerged as essential tools for modulating these processes with precision [2]. A wide array of compounds are employed to enhance rumen fermentation, improve feed digestibility, and bolster overall animal health [3]. Against this backdrop, D-ribose has gathered considerable attention as a potential growth promoter. As a pentose sugar, D-ribose occupies a central role in the pentose phosphate pathway, catalyzing rapid nucleotide synthesis and energy generation [4]. However, its importance transcends these well-documented metabolic roles. Recent microbiological research has unveiled that D-ribose can function as a quorum sensing inhibitor [5]. Quorum sensing (QS) is a sophisticated communication system among rumen microbes that orchestrates their collective behavior, including biofilm formation and fermentation pathways [6]. By interfering with these signaling pathways, D-ribose has the potential to reshape the microbial community structure and function, redirecting fermentation processes toward more host-beneficial outcomes [5]. This dual capacity of D-ribose, serving both as a metabolic precursor and a microbial modulator, makes it a promising candidate for enhancing growth performance in ruminants. A comprehensive elucidation of the synergistic interplay between its biochemical and microbiological effects is imperative for maximizing its potential application in ruminant nutrition.

The rumen microbiome stands as a fundamental pillar of ruminant nutrition, driving the fermentation processes that transform fibrous plant materials into bioavailable nutrients [7]. This intricate microbial ecosystem, encompassing bacteria, protozoa, fungi, archaea, and phages, is indispensable for efficient feed utilization and overall animal health. Its ability to degrade cellulose and hemicellulose into volatile fatty acids (VFA) and microbial protein endows ruminants with a distinct nutritional advantage [8]. Given its central role, a comprehensive understanding of the rumen microbiome is essential for assessing the effectiveness of feed additives. D-ribose has been shown to impact the composition and metabolism of gut microbiota, as demonstrated in studies involving non-ruminant models [9]. These investigations have revealed that D-ribose can modify microbial communities and influence metabolic pathways, particularly those associated with lipid metabolism and gut barrier function [10]. Our prior research has demonstrated that D-ribose holds potential for enhancing growth performance and overall health in ruminants [11]. However, the mechanisms through which D-ribose influences the rumen microbial community remain to be elucidated. Therefore, it is imperative to investigate how D-ribose modulates the rumen microbiome, potentially uncovering its role as a microbial modulator. Elucidating these interactions will provide critical insights into optimizing feed additives, thereby improving ruminant nutrition and performance.

In ruminants, the hindgut microbiota plays a vital role in fermenting fibrous materials that evade rumen digestion and synthesizing essential nutrients, such as vitamins and short-chain fatty acids [12]. Moreover, the fecal microbiota is crucial for maintaining gut health by supporting a balanced immune response and preventing pathogen colonization [13]. As a result, the fecal microbiota serves as a key indicator of overall gastrointestinal health and digestive efficiency. While feed additives are primarily designed to enhance rumen fermentation and improve feed digestibility, their impact on the fecal microbiota can offer further insights into their efficacy [14]. Previous studies have shown that incorporating feed additives, such as rumen-protected glucose and yeast culture, into the diet can notably reshape the fecal microbial profile [15,16]. These changes involve increasing populations of beneficial bacteria, particularly those within the phylum Firmicutes, while reducing potentially detrimental taxa. Additionally, these additives can influence metabolic pathways associated with carbohydrate and amino acid metabolism. Moreover, the rumen microbiota and fecal microbiota exhibit significant overlap and interconnection, suggesting that fecal samples may occasionally be used as surrogates for rumen microbial communities [17]. Dietary D-ribose supplementation has been shown to enhance growth performance and overall health in ruminants [11], but its effects on the rumen and fecal microbiota remain largely unknown. By examining the combined dynamics of rumen and fecal microbial diversity and community structure, we can better understand how D-ribose supplementation affects nutrient utilization and overall health from a microbial perspective. This approach will help elucidate how D-ribose interacts with the gut microbiota and identify its potential role as a microbial modulator.

Therefore, an 80-day feeding trial was conducted to investigate the effects of dietary D-ribose supplementation on the microbial diversity, community composition, and metabolic function of rumen and fecal microbiota in Hu sheep. The hypothesis was that incorporating D-ribose into the diet would alter specific community composition in both the rumen and feces. The findings of this study provide crucial insights into the intestinal microbiota, thereby facilitating a comprehensive evaluation of the potential applications of D-ribose in ruminant production.

## 2. Materials and Methods

### 2.1. Animal Ethics

In this study, all experimental procedures involving animals were rigorously conducted in strict accordance with the guidelines and regulations established by the Institutional Animal Care and Use Committee at Jiangxi Agricultural University (protocol number: JXAULL-20240416).

### 2.2. Experimental Design

A total of 18 female Hu sheep with similar body weights (20.47 ± 0.58 kg) and ages (90.0 ± 0.28 days) were randomly allocated to two groups, each comprising nine animals. One group was fed a standard basal diet (Control), while the other group received the basal diet supplemented with D-ribose at a concentration of 300 mg kg^−1^ of feed on a dry matter basis (D-Ribose). The D-ribose had a purity level of 99% and was supplied by Jiangxi Chengzhi Bioengineering Co., Ltd. (Yingtan, China). Each sheep was housed individually. To ensure uniform distribution, D-ribose was first blended with finely ground corn (1:20, *w*/*w*) to prepare a premix, which was then incorporated into the total mixed ration and mixed for 20 min. The composition and chemical makeup of the basal diet are detailed in Table 1. The trial lasted 80 days, consisting of an initial 20-day adaptation period during which all sheep were fed the basal diet, followed by a 60-day experimental period where the sheep were fed according to their assigned treatments. Throughout the trial, sheep were fed twice daily at 8:00 a.m. and 6:00 p.m., with feed amounts adjusted to ensure 5–10% feed refusal for the subsequent day. Clean drinking water was available to the animals at all times.

### 2.3. Sample Collection and Feed Analysis

Feed samples were collected on three randomly selected days each week, and they were analyzed for crude protein (CP; method 2001.11), ether extract (EE; method 945.16), calcium (method 935.14), and phosphorus (method 942.23) using AOAC International methods [18]. Additionally, neutral detergent fiber (NDF) and acid detergent fiber (ADF) were determined following the procedures of Van Soest et al. [19], which included the use of thermostable *α*-amylase. For seven consecutive days prior to the end of the trial, fecal samples were collected from each sheep at 6-h intervals, resulting in a total of 28 samples per sheep. These samples were subsequently pooled in equal proportions to generate a composite fecal sample representative of each individual sheep. On two days preceding the trial’s conclusion, rumen contents were collected using the oral esophageal tubing method described by Paz et al. [20], prior to the morning feeding. The initial 100 mL of the rumen contents was discarded to minimize salivary contamination. The remaining rumen content was then filtered through four layers of cheesecloth to obtain the rumen fluid samples. Both the fecal and rumen fluid samples were stored at −80 °C for subsequent DNA extraction and sequencing analysis.

### 2.4. DNA Extraction, Sequencing, and Data Analysis

A total of 36 samples, comprising 18 rumen fluid samples and 18 fecal samples, were utilized for total DNA extraction, which was conducted in strict adherence to the manufacturer’s instructions using the specified kits (Omega Bio-tek, Norcross, GA, USA). The V1–V9 regions of the 16S rRNA gene were amplified using primers 27F (5′-AGRGTTYGATYMTGGCTCAG-3′) and 1492R (5′-RGYTACCTTGTTACGACTT-3′). Each sample’s amplification primers incorporated an 8-base tag sequence (Pacific Biosciences, Menlo Park, CA, USA, PN: 102-135-500) for sample differentiation. The PCR reaction mixture consisted of 4 μL of 5x FastPfu Buffer, 2 μL of 2.5 mM dNTPs, 0.8 μL of each primer (5 μM), 0.4 μL of FastPfu Polymerase, 2 μL of template DNA (10 ng), and 10 μL of H_2_O. The PCR program was set as follows: initial denaturation at 95 °C for 5 min; followed by 30 cycles of denaturation at 95 °C for 30 s, annealing at 55 °C for 30 s, and extension at 72 °C for 45 s; with a final extension at 72 °C for 10 min. The amplified products were purified using the AxyPrep DNA Gel Extraction Kit (Axygen Biosciences, Union City, CA, USA) following 2% agarose gel electrophoresis. SMRTbell libraries were prepared from the purified DNA via blunt-end ligation, following the manufacturer’s protocol (Pacific Biosciences). The amplicons were quantified using a Qubit fluorometer (Qubit 4, Thermo Fisher Scientific, Waltham, MA, USA) and pooled in equimolar amounts. The pooled amplicon mixture was used to construct the sequencing library with the SMRTbell Template Prep Kit 3.0 (Pacific Biosciences, Menlo Park, CA, USA, PN: 102-182-700), following the manufacturer’s protocol. The library was sequenced on the PacBio Sequel II platform. All amplicon sequencing was performed by Shanghai Biozeron Biotechnology Co., Ltd. (Shanghai, China). The raw sequencing data have been deposited in the NCBI SRA database under the accession numbers of PRJNA1300601.

PacBio raw reads were processed using SMRT Link v13.0 (≥3 passes, accuracy ≥0.99) and size-filtered (1000–1800 bp). Barcodes/primers were removed with lima v2.9.0, followed by cutadapt v4.0 (trim-left = 19 nt). ASVs were inferred using the q2-pacbio plugin (DADA2 PacBio-mode) in QIIME 2 2023.7 with maxEE = 12, truncQ = 20, and consensus chimera removal. Full-length ASVs were classified with a Naïve Bayes classifier trained on SILVA 138.2 SSURef NR99 (27F–1492R extract) at 70% confidence. The evaluation of alpha diversity encompassed an array of metrics, including richness as gauged by the Chao1 and ACE indices, diversity as determined by the Shannon and Simpson indices, evenness as assessed with Pielou’s evenness index, and phylogenetic diversity as measured by Faith’s phylogenetic diversity (PD) index. The computations for these indices were conducted using R (version 4.3.3). Non-metric multidimensional scaling (NMDS), utilizing the Bray–Curtis distance as a dissimilarity measure, was selected to characterize beta diversity. This analysis was conducted using the vegan package, a community ecology tool available on R-Forge (https://r-forge.r-project.org/, accessed on 5 August 2025). To further assess the similarity in microbial composition between the control and D-ribose groups, analysis of similarities (ANOSIM) was applied, facilitated by the vegan package in R. Linear discriminant analysis effect size (LEfSe) was employed to identify differentially abundant species between the two groups across various taxonomic levels. The analysis began with a non-parametric factorial Kruskal–Wallis sum-rank test to discern features with significant abundance variations and to identify taxa with substantial differences in abundance. Subsequently, LEfSe utilized linear discriminant analysis (LDA) to quantify the influence of each taxonomic group’s abundance on the observed differentiation, with an LDA score threshold of 5.0 indicating significant discrimination between groups. The Phylogenetic Investigation of Communities by Reconstruction of Unobserved States (PICRUSt2, version 2.5.2) program, which utilizes the Kyoto Encyclopedia of Genes and Genomes (KEGG) database, was employed to predict functional shifts within microbial communities across various samples, revealing the impact of D-ribose supplementation on rumen and fecal metabolic pathways.

### 2.5. Statistical Analysis

To evaluate the normality and homoscedasticity of the dataset, the Shapiro–Wilk and Levene tests were employed, respectively. Variables that satisfying both assumptions (*p* > 0.05) were analyzed using an independent-samples t-test analysis to examine differences between the CON and DR groups. In contrast, for variables that failed to meet either assumption (*p* ≤ 0.05) were analyzed using the non-parametric Mann–Whitney U test. The above statistical analyses were performed using SPSS software (version 20, IBM, Chicago, IL, USA), with a significance threshold of 0.05. Effect size was quantified with Cliff’s delta (δ), computed in R (effsize v0.8.1) using 10,000 bias-corrected and accelerated bootstrap iterations.

## 3. Results

### 3.1. Microbial Alpha-Diversity

The effect of dietary D-ribose supplementation on the alpha diversity of both rumen and fecal microbiota is presented in Table 2. Specifically, D-ribose supplementation had no significant influence on key alpha diversity metrics of the rumen microbiota, including Chao1, ACE, Shannon index, Simpson index, Pielou’s evenness, and Faith’s phylogenetic diversity (Faith’s PD) (*p* > 0.05). Similarly, these indices in the fecal microbiota remained unaffected by D-ribose supplementation (*p* > 0.05).

### 3.2. Microbial Community Composition

The effects of dietary D-ribose supplementation on the composition of rumen and fecal microbiota at the phylum level are presented in Table 3 and Table 4, respectively. In the rumen microbiota, the relative abundance of Cyanobacteria was higher in the Control group than in the D-ribose group (*p* < 0.05). Meanwhile, no significant differences were observed between the two groups in other phyla with a relative abundance greater than 0.01% (*p* > 0.05). In the fecal microbiota, the relative abundances of Verrucomicrobia and *Candidatus* Saccharibacteria were significantly lower in the D-ribose group than in the Control group (*p* < 0.05).

The effects of dietary D-ribose supplementation on the composition of rumen and fecal microbiota at the genus level are presented in Table 5 and Table 6, respectively. Regarding the rumen microbiota, the relative abundances of *Herbivorax* and *Faecalibacterium* were significantly higher in the D-ribose group than in the control group (*p* < 0.05). In the fecal microbiota, the relative abundances of *Lawsonibacter* and *Massilioclostridium* were also significantly higher in the D-ribose group than in the control group (*p* < 0.05). In contrast, the relative abundances of *Bifidobacterium* and *Caproiciproducens* were significantly lower in the D-ribose group than in the control group (*p* < 0.05).

### 3.3. Microbial Beta-Diversity

**Figure 1:**
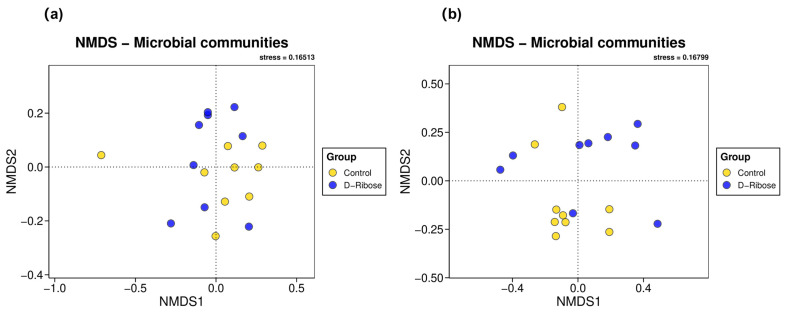
Non-metric multidimensional scaling analysis of (**a**) rumen microbiota and (**b**) fecal microbiota between the group received only the basal diet (Control) and the group received the basal diet supplemented with 300 mg kg^−1^ of D-ribose (D-Ribose).

Figure 1a,b display the NMDS of rumen and fecal microbiota, respectively. The Control group and the D-ribose group exhibited substantial and partial overlap in rumen and fecal microbiota, respectively. ANOSIM of the rumen microbiota revealed no significant differences between the two groups (R = 0.079, *p* = 0.115). In contrast, ANOSIM of the fecal microbiota detected significant differences between the two groups (R = 0.203, *p* = 0.017).

### 3.4. Marked Microbiota

**Figure 2:**
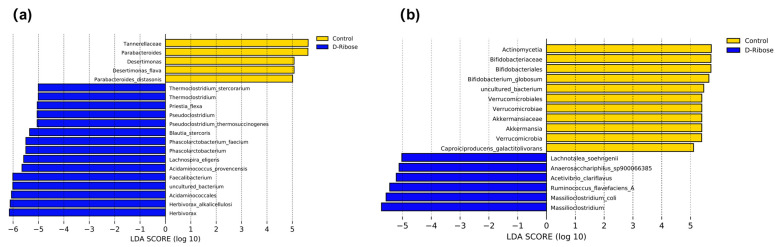
Linear discriminant analysis effect size bar chart of (**a**) rumen microbiota and (**b**) fecal microbiota between the group received only the basal diet (Control) and the group received the basal diet supplemented with 300 mg kg^−1^ of D-ribose (D-Ribose).

LefSe analysis identified microbes present in low abundance at various taxonomic levels. In total, 20 distinct marker microbes were identified in rumen microbiota (Figure 2a). Specifically, the control group was characterized by five distinct marker microbes: Tannerellaceae, *Parabacteroides*, *Desetimonas*, *Desertimonas flava*, and *Parabacteroides distasonis*. The D-ribose group displayed 15 unique marker microbes, including *Thermoclostridium stercorarium*, *Thermoclostridium*, *Priestia flexa*, *Pseudoclostridium*, *Pseudoclostridium thermosuccinogenes*, *Blautia stercoris*, *Phascolarctobacterium faecium*, *Phascolarctobacterium*, *Lachnospira elegants*, *Acidaminococcus provencensis*, *Faecalibacterium*, uncultured bacterium, Acidaminococcales, *Herbivorax alkalicellulosi*, and *Herbivorax*.

Analysis of the fecal microbiota identified 17 distinct marker microbes (Figure 2b). The control group includes several notable microbes such as Actinomycetia, Bifidobacteriaceae, Bifidobacteriales, *Bifidobacterium globosum*, uncultured bacterium, Verrucomicrobiales, Verrucomicrobiae, Akkermansiaceae, *Akkermansia*, Verrucomicrobia, and *Caproiciproducens galactitolivorans*. In contrast, the D-ribose group includes a distinct set of microbes, comprising *Lachnotalea soehngenii*, *Anaerocharis sp900066385*, *Acetivibrio clariflavus*, *Ruminococcus flavefaciens* A, *Massiliclostridium coli*, and *Massiliclostridium*.

### 3.5. Predicted Metabolic Function

**Figure 3:**
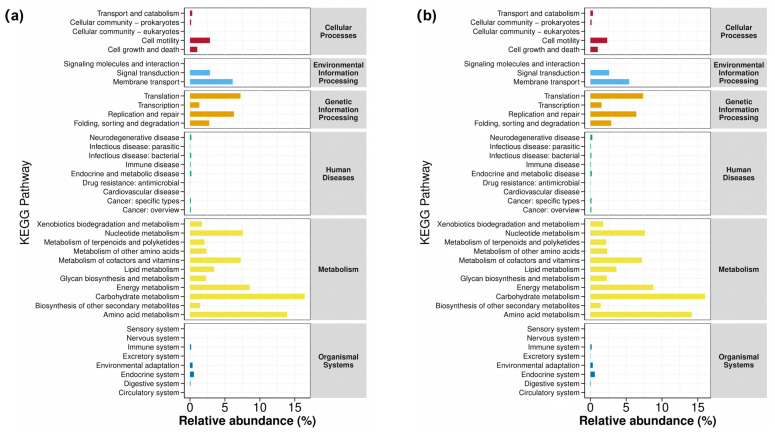
Kyoto Encyclopedia of Genes and Genomes (KEGG) pathway abundance bar chart for metabolic profiling predicted from (**a**) rumen microbiota and (**b**) fecal microbiota.

The effects of dietary D-Ribose supplementation on the relative abundances of predicted metabolic pathways are detailed in Table 7 and Table 8 for rumen and fecal microbiota, respectively. The addition of D-Ribose did not significantly alter the relative abundances of metabolic pathways with relative abundance more than 1% (*p* > 0.05), a finding consistent across both rumen and fecal microbiota. Consistent with this, the top three metabolic pathways in terms of relative abundance in both types of gut microbiota were carbohydrate metabolism, amino acid metabolism, and energy metabolism. Further comparative analysis, as illustrated in Figure 3, revealed that the metabolic functions of the two types of gut microbiota were primarily concentrated in metabolism, genetic information processing, and environmental information processing, with the metabolic pathways within these categories exhibiting similar compositions.

## 4. Discussion

This study revealed that incorporating D-ribose into sheep feed had no effect on the richness, evenness, or diversity of the rumen microbiota, with comparable outcomes observed in fecal microbiota. This lack of impact on microbial diversity may be attributed to the fact that D-ribose, as an energy supplement, primarily influences the host’s metabolic profile [21]. Previous studies in Angus steers have indicated that feed restriction can disrupt nucleotide metabolism and its associated metabolites, which in turn can affect gut barrier function [22]. Beta-diversity analysis further revealed that supplementing the diet with D-ribose had no significant impact on the community structure of the rumen microbiota. This stability of the rumen microbiota might be attributed to the inherent robustness and adaptability of rumen microbial niches. For instance, quorum sensing (QS), a prevalent interspecies communication mechanism within the rumen microbiota, enables microbial communities to collectively respond to adverse environmental shifts [23]. A prime example is the improved concentration of AI-2 signaling molecule and enhanced biofilm formation within the LuxS/AI-2 QS system, which serves as a defense mechanism against the stress induced by cold drinking water during winter [24]. In contrast, the addition of dietary D-ribose did elicit discernible differences in the beta-diversity of fecal microbiota. This discrepancy could stem from the fact that the rumen microbiota is more complex in terms of diversity, composition, and functionality [25], thereby endowing it with superior adaptability to dietary changes than fecal microbiota. Nevertheless, this hypothesis warrants validation through more comprehensive and in-depth comparative investigations.

Species abundance analysis elucidated the effects of dietary D-ribose supplementation on particular microbial taxa in the rumen. The bacteria *Herbivorax*, *Herbivorax alkalicellulosi*, *Thermoclostridium*, *Thermoclostridium stercorarium*, *Priestia flexa*, *Pseudoclostridium*, and *Pseudoclostridium thermosuccinogenes* are all anaerobic and thermophilic, with the ability to break down cellulose [26,27]. The relative abundances of these bacteria in the D-ribose group increased significantly. This implies that incorporating D-ribose into the diet could potentially enhance the rumen’s ability to utilize fiber more effectively, which corresponds well to the higher values of the apparent digestibility of NDF and ADF observed after the addition of D-ribose [11]. *Faecalibacterium*, *Blautia stercoris*, *Phascolarctobacterium*, *Phascolarctobacterium faecium*, and *Lachnospira elegans*, as well as *Lawsonibacter*, Lachnotalea soehngenii, Anaerocharis sp900066385, *Acetivibrio clariflavus*, *Ruminococcus flavefaciens* A, *Massiliclostridium*, and *Massiliclostridium coli* are all strongly associated with gut health. These microorganisms can ferment carbohydrates to generate short-chain fatty acids (SCFA), including acetate, propionate, and butyrate, which are vital for maintaining the integrity of the intestinal barrier and modulating the host’s immune responses [28,29,30]. Meanwhile, Acidaminococcales and *Acidaminococcus provencensis* possess the capability to degrade amino acids and contribute to protein catabolism and metabolism, thus aiding in the maintenance of gut homeostasis [31]. The elevated relative abundance of these microorganisms in the D-ribose group suggests that incorporating D-ribose into the diet enhances the utilization of energy and protein, as evidenced by higher numerical values of EE and CP digestibility [11], which in turn positively impacts rumen and hindgut health. The family Tannerellaceae, as well as the genera *Parabacteroides* and *Desetimonas*, and the species *Parabacteroides distasonis* and *Desertimonas flava*, are associated with various intestinal diseases and inflammatory responses [32,33]. For instance, succinate, a metabolite produced by *Parabacteroides distasonis*, can act as an inflammatory signal. It induces the production of IL-1*β* via the HIF-1*α* pathway, thereby triggering an inflammatory response. This, in turn, disrupts the delicate balance of the gut microbiota and negatively impacts nutrient absorption and metabolism [32]. In this study, an increased relative abundance of these microorganisms in the control group indirectly suggests that the addition of D-ribose can enhance the overall health status of the host. These differences observed in the rumen microbial composition further indicate that incorporating D-ribose into the diet can improve the utilization efficiency of energy, protein, and fiber, as well as positively impact the host’s overall health, which is in line with our previous research findings that dietary D-ribose supplementation improves nutrient digestion and reduces stress responses [11].

The analysis of fecal differential microbiota more deeply assessed the impact of dietary D-ribose supplementation on both the composition and the dominant communities of the fecal microbiota. *Candidatus* Saccharibacteria remains an elusive microorganism, yet to be successfully cultured in laboratory settings. Its elevated abundance has been linked to gut microbiota dysbiosis in individuals suffering from inflammatory bowel diseases, such as Crohn’s disease [34]. Similarly, an increase in the prevalence of Verrucomicrobia, along with its associated classes Verrucomicrobiae and orders Verrucomicrobiales, may serve as a telltale sign of gut microbiota imbalance, potentially foreshadowing the onset of disease [35]. The lower abundances of these fecal microorganisms observed in the D-ribose group suggest that the incorporation of D-ribose into the diet may play a pivotal role in preserving the stability of the gut microbiota, thereby fostering a healthier gastrointestinal environment. Bifidobacteriales, along with Bifidobacteriaceae, *Bifidobacterium*, and *Bifidobacterium globosum*, are generally recognized as beneficial bacteria. They modulate the host immune system and boost the activity of regulatory T cells. These bacteria also enhance the barrier function of intestinal epithelial cells, thereby reducing intestinal permeability [36]. The metabolites they produce, such as acetate and butyrate, help maintain the balance of the gut microbiota [36]. Actinomycetia are prevalent in soil, water, and living organisms. They are significant producers of secondary metabolites, including antibiotics and bioactive substances, which play a crucial role in inhibiting pathogenic bacteria and maintaining ecological balance [37]. Surprisingly, in this study, the abundance of these microorganisms in the feces of the control group was significantly higher. One possible explanation is that both the control and D-ribose groups remained healthy throughout the experiment, with no abnormalities in fecal status. The fecal microbiota may exhibit functional redundancy, where other microorganisms can compensate for the reduced abundance of certain species, thereby maintaining overall health [38]. Alternatively, in the D-ribose group, these microorganisms may have been outcompeted by other beneficial microorganisms, leading to a more stable microbial ecological balance. This finding underscores the need for absolute quantification of microbial composition to determine absolute abundance, rather than relying solely on relative abundance data from conventional techniques. *Caproiciproducens* and *Caproiciproducens galactitolivorans* are recognized for their ability to ferment substrates such as fructose, lactate, galactose, and xylose, resulting in the production of SCFA [39]. Similarly, Akkermansiaceae and *Akkermansia* are known for degrading mucin in the gut, which also leads to the generation of SCFA [40]. While these metabolic activities are typically linked to gut health, emerging research indicates that an increased abundance of these microorganisms can be observed in the context of gut dysbiosis, particularly during *Salmonella* infections [41]. The differential fecal microbial results suggest that dietary D-ribose supplementation specifically boosts the abundance of beneficial microorganisms associated with immune metabolism. This effect contrasts sharply with the impact on the rumen microbiota, where the primary microorganisms drive nutrient digestion and utilization.

D-ribose is a fundamental component in nucleotide metabolism, playing a crucial role in various biological processes, including nucleotide synthesis, energy metabolism, signal transduction, antioxidant defense, DNA repair, and metabolic regulation. However, the predicted metabolic pathways in this study did not reveal any differences in the aforementioned metabolic pathways between the CON and D-ribose groups. Measuring the nucleotide content in blood and rumen fluid could better reveal the relationship between nucleotide metabolism and microbial responses. Moreover, integrating host data with microbial data could provide a more comprehensive explanation for the dynamics of rumen and fecal microbiota due to dietary D-ribose supplementation.

It should be acknowledged that the present investigation exclusively evaluated the impact of dietary D-ribose supplementation at 300 mg kg^−1^ on ruminal and fecal microbial communities under non-stress conditions. Consequently, whether lower or otherwise alternative doses elicit comparable responses remains to be established. Under environmental challenges such as heat stress, cold stress, or transportation stress, the ruminal microbiota of ruminants undergoes marked compositional and functional shifts; accordingly, the optimal supplemental level of D-ribose and its resultant efficacy under such circumstances awaits systematic clarification in future research. Furthermore, the functional predictions presented here were generated with PICRUSt2, which was trained primarily on human-microbiome data; therefore, the absence of significant differences should be interpreted cautiously and verified in future studies using rumen-specific tools such as CowPI or direct metagenomics.

## 5. Conclusions

Dietary D-ribose supplementation did not alter the alpha diversity or metabolic functions of the rumen and fecal microbiota. However, it impacted the beta diversity of fecal microbiota and influenced the community composition of specific rumen and the dominants communities of fecal microbiota. Based on the changes in the dynamics of the rumen and fecal microbiota, dietary R-ribose supplementation with 300 mg kg^−1^ in Hu sheep is an option for a feed additive under the conditions in which the study was carried out.

## Figures and Tables

**Table 1:** Ingredients and nutrient composition of the basal diet.

Ingredient	Proportion, %
Corn	25.00
Soybean meal	10.50
Wheat bran	12.75
Wheat straw	28.75
Peanut straw	20.00
Calcium hydrogen phosphate	1.00
Limestone	0.50
Salt	0.50
Premix ^1^	1.00
**Chemical composition**	Value
Metabolizable energy ^2^, MJ kg^−1^	10.06
Crude protein, g kg^−1^	130.6
Neutral detergent fiber, g kg^−1^	402.0
Acid detergent fiber, g kg^−1^	245.6
Ether extract, g kg^−1^	26.7
Calcium, g kg^−1^	8.7
Phosphorus, g kg^−1^	5.0

^1^ Premix provided the following per kg of DM: 1400 mg of Fe, 1200 mg of Zn, 250 mg of Cu, 900 mg of Mn, 100,000 IU of vitamin A, 27,000 IU of vitamin D3, and 800 IU of vitamin E. ^2^ The metabolizable energy (ME) value of the feed was estimated by summing the products obtained from multiplying the ME values of each feed ingredient by their respective proportions.

**Table 2:** Effect of dietary D-ribose supplementation on the alpha-diversity of rumen and fecal microbiota.

Item	Control	D-Ribose	SEM	*p*-Value	Effect Size
**Rumen**					
Chao1	4459.11	4747.22	126	0.126	−0.383
ACE	4459.11	4747.28	126	0.126	−0.383
Shannon index	7.46	7.80	0.160	0.436	−0.235
Simpson index	0.9839	0.9957	0.004	0.436	−0.235
Pielou’s evenness	0.8878	0.9210	0.017	0.489	−0.210
Faith’s PD	280.20	292.22	5.59	0.149	−0.358
**Feces**					
Chao1	5145.11	5460.67	173	0.214	−0.321
ACE	5145.16	5460.69	173	0.214	−0.333
Shannon index	8.31	8.23	0.075	0.546	−0.185
Simpson index	0.9993	0.9978	0.001	0.546	0.185
Pielou’s evenness	0.9728	0.9568	0.007	0.258	0.333
Faith’s PD	309.21	320.55	7.05	0.272	−0.358

Control, the group received only the basal diet; D-Ribose, the group received the basal diet supplemented with 300 mg kg^−1^ of D-ribose; SEM, standard error of the mean.

**Table 3:** Effects of dietary D-ribose supplementation on the composition of rumen microbiota at the phylum level.

Phylum Name	Control	D-Ribose	SEM	*p*-Value	Effect Size
Firmicutes	68.84	75.89	4.94	0.349	−0.185
Bacteroidetes	17.70	13.90	4.20	0.730	0.111
Actinobacteria	10.34	7.65	3.62	0.863	−0.062
Proteobacteria	2.33	2.05	0.201	0.489	0.210
Planctomycetes	0.52	0.13	0.176	0.222	0.358
Acidobacteria	0.09	0.15	0.021	0.059	−0.506
Synergistetes	0.02	0.10	0.053	0.436	0.235
Verrucomicrobia	0.04	0.02	0.010	0.387	0.259
*Candidatus* Saccharibacteria	0.04	0.02	0.011	0.730	0.111
Tenericutes	0.01	0.03	0.013	0.665	0.123
Cyanobacteria	0.03	0.002	0.005	0.014	0.679
Spirochaetes	0.004	0.02	0.007	0.136	−0.432
Gemmatimonadetes	0.02	0.01	0.005	0.297	0.296

Control, the group received only the basal diet; D-Ribose, the group received the basal diet supplemented with 300 mg kg^−1^ of D-ribose; SEM, standard error of the mean.

**Table 4:** Effects of dietary D-ribose supplementation on the composition of fecal microbiota at the phylum level.

Phylum Name	Control	D-Ribose	SEM	*p*-Value	Effect Size
Firmicutes	62.07	65.55	3.26	0.475	−0.309
Bacteroidetes	29.23	26.51	3.15	0.586	0.383
Proteobacteria	2.63	3.66	0.479	0.436	−0.235
Spirochaetes	1.89	2.37	0.381	0.394	−0.259
Actinobacteria	2.77	1.28	0.473	0.050	0.556
Verrucomicrobia	1.09	0.20	0.158	0.014	0.679
Fibrobacteres	0.07	0.21	0.052	0.489	−0.210
Acidobacteria	0.09	0.10	0.021	0.610	−0.160
Planctomycetes	0.09	0.04	0.018	0.436	0.235
*Candidatus* Saccharibacteria	0.04	0.01	0.007	0.040	0.580
Elusimicrobia	0.01	0.03	0.008	0.136	−0.420
Cyanobacteria	0.01	0.01	0.006	0.605	−0.160

Control, the group received only the basal diet; D-Ribose, the group received the basal diet supplemented with 300 mg kg^−1^ of D-ribose; SEM, standard error of the mean.

**Table 5:** Effects of dietary D-ribose supplementation on the composition of rumen microbiota at the genus level.

Genus Name	Control	D-Ribose	SEM	*p*-Value	Effect Size
*Prevotella*	10.98	9.34	3.04	0.931	−0.037
*Mitsuokella*	10.50	2.13	3.29	0.340	0.284
*Butyrivibrio*	5.26	6.23	1.67	0.796	−0.086
*Lachnospira*	4.77	5.45	2.17	0.436	−0.235
*Bifidobacterium*	6.10	1.27	1.99	0.666	0.136
*Enterocloster*	2.71	4.59	0.775	0.161	−0.407
*Eisenbergiella*	2.50	3.61	0.546	0.436	−0.235
*Herbivorax*	0.84	4.69	1.12	0.019	−0.654
*Acetivibrio*	1.91	3.59	1.23	0.340	−0.284
*Selenomonas*	3.16	1.90	1.56	0.094	0.481
*Pseudoscardovia*	2.21	2.77	1.98	0.136	−0.432
*Olsenella*	1.51	3.28	1.17	0.161	−0.407
*Caproiciproducens*	4.50	0.28	2.21	0.666	0.136
*Blautia*	1.42	2.55	0.520	0.190	−0.383
*Agathobacter*	2.32	1.43	0.650	0.730	0.111
*Succiniclasticum*	0.95	2.65	0.592	0.113	−0.457
*Ruminococcus*	2.14	1.43	0.802	0.796	0.086
*Faecalibacterium*	0.64	2.53	1.03	0.024	−0.630
*Parabacteroides*	2.39	0.77	0.543	0.094	0.481
*Sodaliphilus*	1.64	1.29	0.574	0.605	0.160
*Roseburia*	1.43	1.40	0.325	0.863	−0.062
*Christensenella*	1.08	1.43	0.371	0.730	−0.111
*Emergencia*	0.82	1.20	0.219	0.340	−0.284

Control, the group received only the basal diet; D-Ribose, the group received the basal diet supplemented with 300 mg kg^−1^ of D-ribose; SEM, standard error of the mean.

**Table 6:** Effects of dietary D-ribose supplementation on the composition of fecal microbiota at the genus level.

Genus Name	Control	D-Ribose	SEM	*p*-Value	Effect Size
*Rikenella*	6.57	8.13	2.27	0.546	0.185
*Bacteroides*	5.31	4.41	0.574	0.288	0.333
*Christensenella*	5.39	3.91	0.710	0.387	0.259
*Papillibacter*	4.29	4.73	0.483	0.539	−0.259
*Muribaculum*	4.65	4.23	0.540	0.606	0.259
*Ruminococcus*	2.86	4.57	0.624	0.190	−0.383
*Lawsonibacter*	3.14	3.79	0.202	0.038	−0.531
*Oscillibacter*	3.55	2.91	0.378	0.255	0.407
*Phocaeicola*	2.98	2.40	0.425	0.931	−0.037
*Acetatifactor*	2.02	3.30	0.433	0.075	−0.383
*Duncaniella*	3.74	1.30	0.700	0.113	0.457
*Intestinimonas*	2.41	2.60	0.233	0.588	−0.185
*Massilioclostridium*	1.51	3.33	0.394	0.004	−0.778
*Dysosmobacter*	2.51	2.17	0.385	0.560	0.407
*Acetivibrio*	2.23	2.36	0.300	1.000	−0.012
*Treponema*	1.54	2.14	0.280	0.161	−0.432
*Anaerostipes*	1.26	2.36	0.677	0.546	0.185
*Alistipes*	1.78	1.68	0.124	0.611	0.136
*Prevotella*	1.59	1.65	0.302	0.891	0.012
*Flavonifractor*	1.47	1.58	0.287	0.436	0.235
*Bifidobacterium*	2.03	0.68	0.435	0.031	0.605
*Monoglobus*	1.18	1.39	0.191	0.472	−0.086
*Faecalimonas*	1.17	1.39	0.148	0.666	−0.136
*Caproiciproducens*	1.62	0.78	0.159	0.002	0.778
*Eisenbergiella*	1.06	1.21	0.248	0.673	−0.160
*Kineothrix*	0.86	1.34	0.206	0.222	−0.358
*Blautia*	1.02	1.04	0.287	0.796	0.086

Control, the group received only the basal diet; D-Ribose, the group received the basal diet supplemented with 300 mg kg^−1^ of D-ribose; SEM, standard error of the mean.

**Table 7:** Effect of dietary D-ribose supplementation on the relative abundance of the predicted metabolic pathways in the rumen bacterial microbiome of Hu sheep.

Metabolic Pathway	Control	D-Ribose	SEM	*p*-Value	Effect Size
Carbohydrate metabolism	16.48	16.36	0.146	0.576	0.160
Amino acid metabolism	13.94	13.86	0.086	0.574	0.136
Energy metabolism	8.62	8.45	0.093	0.222	0.309
Nucleotide metabolism	7.53	7.59	0.048	0.376	−0.185
Metabolism of cofactors and vitamins	7.20	7.29	0.133	0.642	−0.062
Translation	7.21	7.24	0.068	0.785	−0.210
Replication and repair	6.26	6.33	0.044	0.314	−0.235
Membrane transport	6.03	6.19	0.193	0.582	−0.160
Lipid metabolism	3.44	3.47	0.061	1.000	0.012
Signal transduction	2.80	2.92	0.108	0.458	−0.185
Cell motility	2.78	2.92	0.233	0.692	−0.062
Folding, sorting and degradation	2.74	2.77	0.027	0.359	−0.210
Metabolism of other amino acids	2.37	2.30	0.033	0.194	0.383
Glycan biosynthesis and metabolism	2.30	2.24	0.080	0.627	0.136
Metabolism of terpenoids and polyketides	2.11	2.05	0.034	0.436	0.235
Xenobiotics biodegradation and metabolism	1.77	1.63	0.046	0.077	0.506
Biosynthesis of other secondary metabolites	1.46	1.44	0.032	0.632	0.136
Transcription	1.28	1.38	0.045	0.125	−0.457
Cell growth and death	1.04	1.04	0.010	0.732	0.210

Control, the group received only the basal diet; D-Ribose, the group received the basal diet supplemented with 300 mg kg^−1^ of D-ribose; SEM, standard error of the mean.

**Table 8:** Effect of dietary D-ribose supplementation on the relative abundance of the predicted metabolic pathways in the fecal bacterial microbiome of Hu sheep.

Metabolic Pathway	Control	D-Ribose	SEM	*p*-Value	Effect Size
Carbohydrate metabolism	16.04	15.99	0.083	0.691	0.037
Amino acid metabolism	14.16	14.13	0.072	0.222	0.358
Energy metabolism	8.81	8.78	0.045	0.674	0.284
Nucleotide metabolism	7.62	7.59	0.029	0.536	−0.136
Translation	7.36	7.36	0.033	0.988	−0.086
Metabolism of cofactors and vitamins	7.22	7.18	0.043	0.666	0.235
Replication and repair	6.41	6.41	0.022	0.938	−0.062
Membrane transport	5.40	5.46	0.149	0.787	−0.333
Lipid metabolism	3.65	3.63	0.023	0.480	0.259
Folding, sorting and degradation	2.89	2.92	0.014	0.200	−0.383
Signal transduction	2.57	2.66	0.085	0.507	−0.407
Metabolism of other amino acids	2.36	2.35	0.024	0.608	0.259
Cell motility	2.28	2.42	0.103	0.355	−0.333
Glycan biosynthesis and metabolism	2.32	2.27	0.034	0.281	0.358
Metabolism of terpenoids and polyketides	2.21	2.22	0.022	0.297	0.309
Xenobiotics biodegradation and metabolism	1.79	1.76	0.034	0.340	0.284
Transcription	1.55	1.58	0.020	0.260	−0.309
Biosynthesis of other secondary metabolites	1.46	1.43	0.020	0.383	0.259
Cell growth and death	1.04	1.04	0.009	0.824	0.160

Control, the group received only the basal diet; D-Ribose, the group received the basal diet supplemented with 300 mg kg^−1^ of D-ribose; SEM, standard error of the mean.

## Data Availability

The raw sequencing data presented in this study are openly available in the NCBI SRA database under the accession numbers of PRJNA1300601.

## Abbreviations

The following abbreviations are used in this manuscript:

QS	Quorum sensing
VFA	Volatile fatty acids
PD	Phylogenetic diversity
NMDS	Non-metric multidimensional scaling
ANOSIM	Analysis of similarities
LEfSe	Linear discriminant analysis effect size
LDA	Linear discriminant analysis
PICRUSt	Phylogenetic Investigation of Communities by Reconstruction of Unobserved States
KEGG	Kyoto Encyclopedia of Genes and Genomes
SCFA	Short-chain fatty acids
